# Plant Metabolites as SARS-CoV-2 Inhibitors Candidates: In Silico and In Vitro Studies

**DOI:** 10.3390/ph15091045

**Published:** 2022-08-24

**Authors:** Alberto Jorge Oliveira Lopes, Gustavo Pereira Calado, Yuri Nascimento Fróes, Sandra Alves de Araújo, Lucas Martins França, Antonio Marcus de Andrade Paes, Sebastião Vieira de Morais, Cláudia Quintino da Rocha, Cleydlenne Costa Vasconcelos

**Affiliations:** 1 Federal Institute of Science Education and Technology of Maranhão—Campus Santa Inês, Castelo Branco, s/n—Canaã, Santa Inês 65300-000, Brazil; 2 Programa de Pós-graduação em Ciências Farmacêuticas—PPGCF, Departamento de Farmácia, Universidade de Brasília-UnB Brasília-DF, Brasilia 70910-900, Brazil; 3 Laboratory of Microbial Pathogenicity, CEUMA Univesity, São Luís 65075-120, Brazil; 4 Programa de Pós-graduação em Biotecnologia/RENORBIO, Universidade Federal do Maranhão, São Luís 65080-805, Brazil; 5 Physiological Sciences Department, Federal University of Maranhão, São Luís 65080-805, Brazil; 6 Chemistry Department, Federal University of Maranhão, São Luís 65080-805, Brazil

**Keywords:** new drugs agents, medicinal chemistry, in silico protocols, natural products

## Abstract

Since it acquired pandemic status, SARS-CoV-2 has been causing all kinds of damage all over the world. More than 6.3 million people have died, and many cases of sequelae are in survivors. Currently, the only products available to most of the world’s population to fight the pandemic are vaccines, which still need improvement since the number of new cases, admissions into intensive care units, and deaths are again reaching worrying rates, which makes it essential to compounds that can be used during infection, reducing the impacts of the disease. Plant metabolites are recognized sources of diverse biological activities and are the safest way to research anti-SARS-CoV-2 compounds. The present study computationally evaluated 55 plant compounds in five SARS-CoV-2 targets such Main Protease (Mpro or 3CL or MainPro), RNA-dependent RNA polymerase (RdRp), Papain-Like Protease (PLpro), NSP15 Endoribonuclease, Spike Protein (Protein S or Spro) and human Angiotensin-converting enzyme 2 (ACE-2) followed by in vitro evaluation of their potential for the inhibition of the interaction of the SARS-CoV-2 Spro with human ACE-2. The in silico results indicated that, in general, amentoflavone, 7-O-galloylquercetin, kaempferitrin, and gallagic acid were the compounds with the strongest electronic interaction parameters with the selected targets. Through the data obtained, we can demonstrate that although the indication of individual interaction of plant metabolites with both Spro and ACE-2, the metabolites evaluated were not able to inhibit the interaction between these two structures in the in vitro test. Despite this, these molecules still must be considered in the research of therapeutic agents for treatment of patients affected by COVID-19 since the activity on other targets and influence on the dynamics of viral infection during the interaction Spro x ACE-2 should be investigated.

## 1. Introduction

The new coronavirus severe acute respiratory syndrome (syn. SARS-CoV-2) has rapidly evolved to pandemic status and is being reported rapidly around the world. Since the beginning of the outbreak in China, more than 594 million cases and 6.4 million deaths have been reported worldwide (“Coronavirus Disease (COVID-19) Situation Reports,” [n.d.]), and more than 10% of the deaths occurred in Brazil.

Although the infection was initially classified as zoonotic, during the pandemic it became clear that human-to-human infection was the most frequent model of transmission [1], occurring through the deliberate release of aerosols from SARS-CoV-2 carriers, mainly in closed environments through the upper airways or ocular mucosa [2,3].

Fever and cough are the most common symptoms, followed by fatigue, sputum production, and shortness of breath [4]. The groups most susceptible to having severe acute respiratory syndrome and various complications of COVID-19 are the elderly and those with metabolic syndromes (diabetes, hypertension, and cardiovascular diseases). If not treated in time, they may progress to severe acute respiratory syndrome, septic shock, and general haematological disorders (coagulation and metabolic acidosis), leading to death within days to a few days [4,5].

The complications of COVID-19 have an aggressive inflammatory character, and corticoids are the main tools used. The use of corticosteroids in patients with COVID-19 pneumonia may modulate the inflammatory response, thereby reducing the risk of the infected developing an acute respiratory syndrome (ARS), however the use of corticoids presents several side effects, especially in cases of prolonged use in hospitalized patients [6,7,8,9,10].

In the absence of approved effective drug therapies and vaccines, space opens for screening new drugs for preclinical studies. Numerous studies have demonstrated the importance of natural product bioprospecting and molecular docking as alternative tools for the search of efficient compounds to combat COVID-19 [7,11,12,13]. The in silico protocols provide a more promising direction for research involving the study and planning of new drugs with numerous cases of success in various areas of knowledge [14,15,16], due they offer important information about the interactions between molecules and their targets of interest, presenting expressive results in pharmacological experiments, favouring the experimental design of pre-clinical trials, allowing more promising experimental approaches [17,18,19]. In this perspective, computational methodologies have been employed for the identification and selection of potential new drugs. This approach allows for subsidizing the planning and development of bioactive compounds, suggesting which of these compounds may be used in the prevention, treatment, or cure of diseases [14,15,20,21].

In silico techniques achieve success in identifying and selecting molecules with bioactive potential as human immunodeficiency virus (HIV) protease inhibitors [22,23,24]. Other advances are the anti-inflammatory drugs specific inhibitors of the cyclooxygenase-2 (COX-2) enzyme [25,26], the antigripal Relenza™, specific inhibitor of the viral neuraminidase enzyme [27] and the anticancer drug Glivec™, specific inhibitor of protein tyrosine kinase [28,29], in addition to tirofiban, a fibrinogen antagonist [30], saquinavir, ritonavir and indinavir, drugs for the treatment of HIV [31], dorzolamide, a carbonic anhydrase inhibitor [32] and the antihypertensive captopril [33].

When there is a large library of compounds to be evaluated, the classification of these compounds provided by docking, brings a valuable aid in the identification of new drug candidates, directing the subsequent experimental research for drug development [34], allowing a substantial economy of financial resources and enabling the achievement of effective results, which would allow the research to evolve more quickly to subsequent steps.

In silico, therapeutic targets such as Main Protease (Mpro or 3CL or MainPro), RNA-dependent RNA polymerase (RdRp), Papain-Like Protease, NSP15 Endoribonuclease, Spike Protein (or Protein S or Spro) and Angiotensin-converting enzyme 2 (ACE-2) [7], can be tested in silico with phytoconstituents to evaluate the possible reduction of viral adsorption or even reduce viral replication, entailing new candidates for in vitro and in vivo studies.

This study, based on previous data from our studies of anti-inflammatory plant activity, aimed to evaluate plant metabolites by in silico protocols against SARS-CoV-2 targets and to evaluate them in vitro for their potential to inhibit the interaction of SARS-CoV-2 Spro with human ACE-2, thus allowing the prioritization of classes or compounds for further prospecting studies based on these natural products and providing new data about the dynamics of inhibition of virus infection in the host cell.

## 2. Results

### 2.1. Molecular Docking

**Figure 1:**
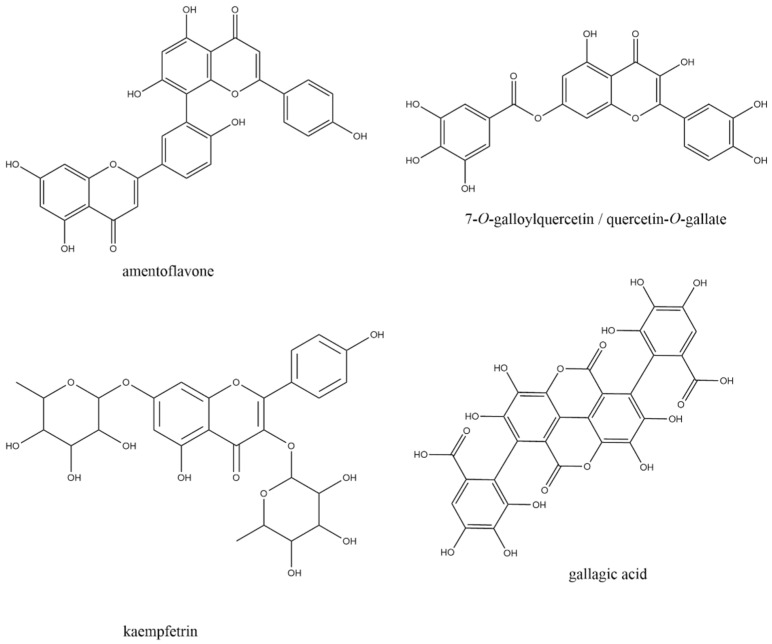
Schematic representation of the four plant metabolites that presented the most promising interaction parameters with SARS-CoV-2 drug targets, through molecular docking.

After geometric, electronic, and spatial optimization by Density Functional Theory (DFT) studies, the 55 plant metabolites selected were evaluated in the six SARS-CoV-2 chosen targets, resulting in a total of 330 molecular docking calculations. In all targets evaluated, the best electronic affinity parameters, defined by the Gibbs free binding energy* (∆Gbind; in kcal/mol), were presented by polyphenols and flavonoids amentoflavone, 7-O-galloylquercetin, kaempferitrin, and gallagic acid (Figure 1), for being ranked among the five compounds with the best electronic affinity parameters in at least four of the six selected targets (Table 1).

To validate the molecular docking protocol, the re-docking of native ligands of crystal structures selected was carried out. As for the Root Mean Square Deviation (RMSD) of the redocking of the native ligands of the targets, when these were present, the values were all below 1.76 Å. Values below 2 Å are reported in the literature as indicative of the accuracy and reliability of the protocol.

**Figure 2:**
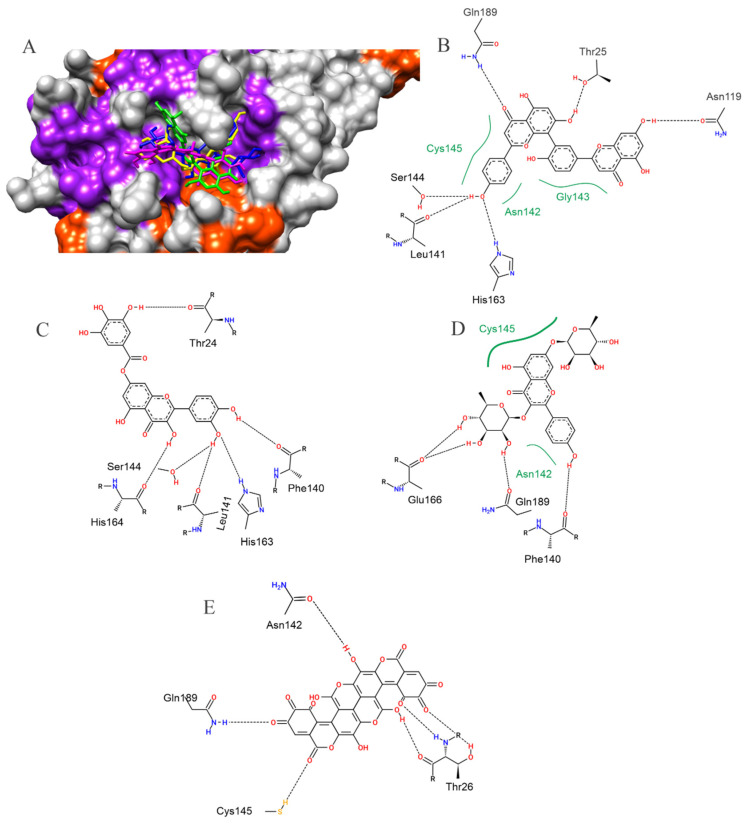
The surface representation of plant metabolites docking positions on the SARS-CoV-2 Mpro active site is shown, with amentoflavone (green), kaempferitrin (blue), 7-O-galloylquercetin (yellow), and gallagic acid (magenta) (**A**). Contacts of SARS-CoV-2 Mpro active site residues with amentoflavone (**B**), 7-O-galloylquercetin (**C**), kaempferitrin (**D**), and gallagic acid (**E**) are depicted in a two-dimensional diagram. Dashed black lines indicate hydrogen bonds; full green lines indicate van der Waals interactions.

For the SARS-CoV-2 main protease (Mpro), the best affinity parameters were presented by amentoflavone (−8.7 kcal/mol), followed by 7-o-galloylquercetin (−8.6 kcal/mol), kaempferitrin (−8.6 kcal/mol), digalloylshikimic acid (−8.4 kcal/mol) and gallagic acid (−8.2 kcal/mol) (Table 1). It is observed that all selected metabolites presented hydrogen bond-type interactions with important residues of the active site (Cys145) and with residues in the vicinity of this region (Figure 2).

**Figure 3:**
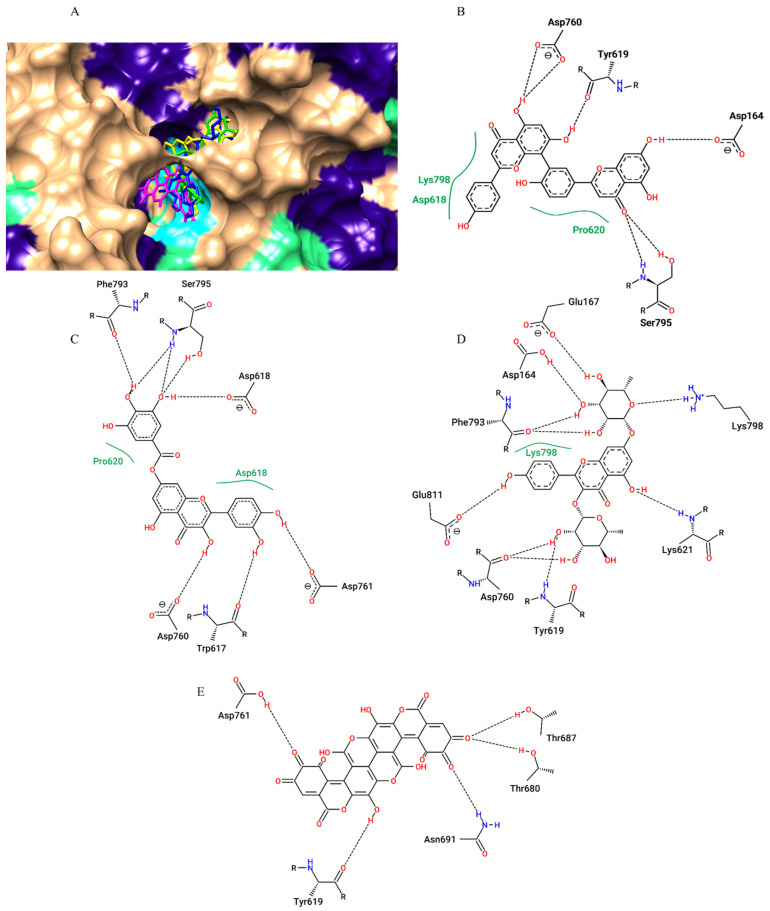
Surface representation of plant metabolites docking positions on the SARS-CoV-2 RdRp active site, with amentoflavone (green), kaempferitrin (blue), 7-O-galloylquercetin (yellow), and gallagic acid (magenta) (**A**). The two-dimensional diagram from contacts of SARS-CoV-2 RDRP active site residues with amentoflavone (**B**), 7-O-galloylquercetin (**C**), kaempferitrin (**D**) and gallagic acid (**E**) Dashed black lines indicate hydrogen bonds; full green lines indicate van der Waals interactions.

For the SARS-CoV-2 RNA-dependent RNA polymerase (RdRp), the best affinity parameters were presented by amentoflavone (−9.4 kcal/mol), followed by kaempferitrin (−9.3 kcal/mol), gallagic acid (−9.0 kcal/mol), 7-O-galloylquercetin (−8.8 kcal/mol) and typhaneoside (−8.6 kcal/mol) (Table 1). All the selected metabolites showed hydrogen bond type interactions and van der Walls interactions with important residues of the active site (Asp618) and with residues in the vicinity of this region (Figure 3).

**Figure 4:**
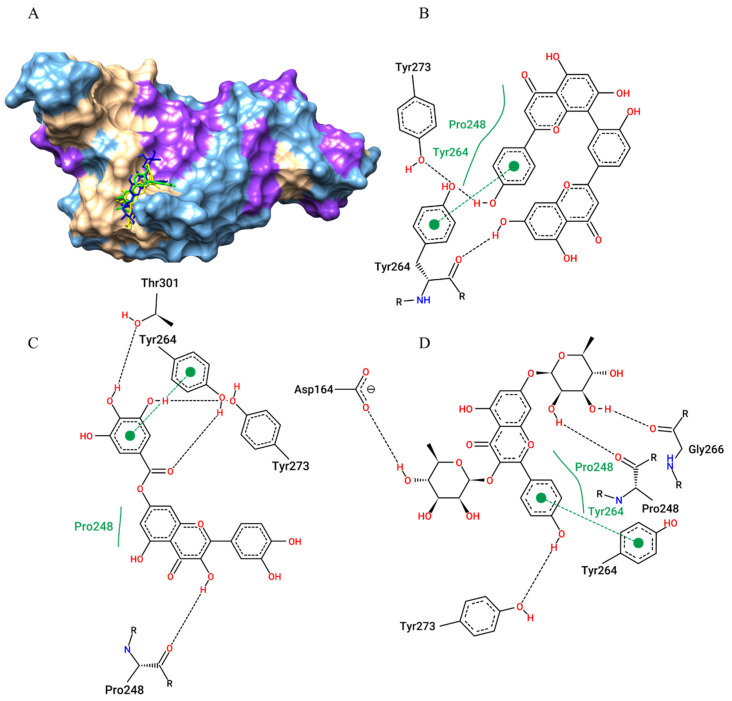
Amentoflavone (green), kaempferitrin (blue), and 7-O-galloylquercetin (yellow) docking positions on the SARS-CoV-2 Papain-like protease (PLpro) active site are shown on the surface (**A**). Contacts of SARS-CoV-2 PLpro active site residues with amentoflavone (**B**), 7-O-galloylquercetin (**C**), and kaempferitrin (**D**) are depicted in a two-dimensional diagram. Dashed black lines represent hydrogen bonds; full green lines represent van der Waals interactions; and dashed green lines represents π-π stacking.

In the molecular docking study of the 55 metabolites with the SARS-CoV-2 Papain-like protease (PLpro), the best electronic affinity parameters were amentoflavone (−7.7 kcal/mol), kaempferitrin (−7.5 kcal/mol), 7-O-galloylquercetin (−7.4 kcal/mol) and myricitrin and myricetin (both −7.3 kcal/mol) (Table 1). We also showed that the protocol worked well in predicting the position of the ligands because they performed their interactions (hydrogen bonds, van der Walls interactions, and π-π stacking with Tyr264, the active site residue, as well as with neighbouring residues (Figure 4). 

**Figure 5:**
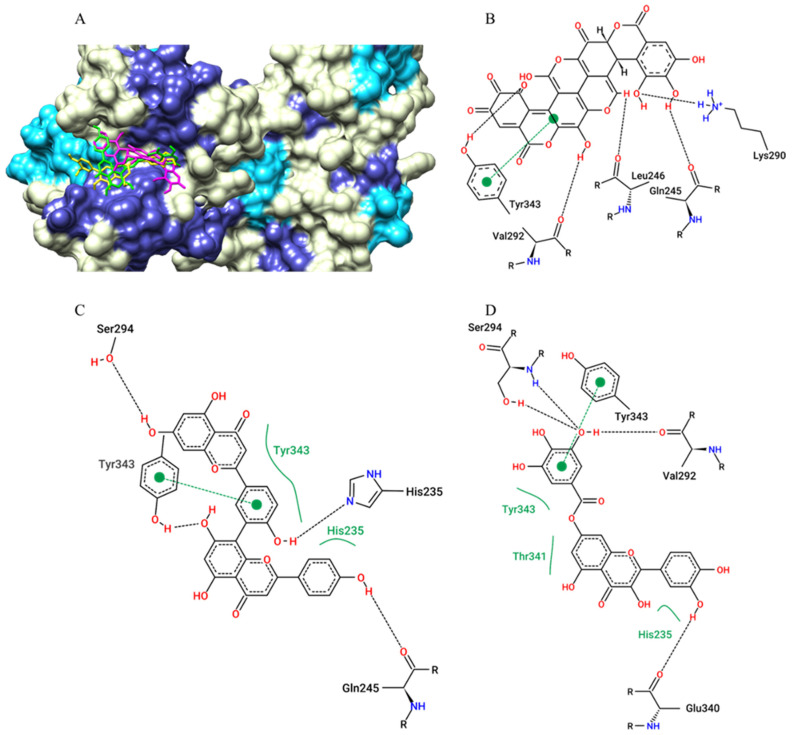
Surface representation of plant metabolites docking positions on the SARS-CoV-2 NSP15 endoribonuclease active site, with amentoflavone (green), 7-O-galloylquercetin (yellow), and gallagic acid (magenta) (**A**). Contacts of SARS-CoV-2 NSP15 endoribonuclease active site residues with gallagic acid (**B**), amentoflavone (**C**), and 7-O-galloylquercetin (**D**) are depicted in two dimensions. Dashed black lines represent hydrogen bonds, while full green lines represent van der Waals interactions. dashed green line represent π-π stacking.

For SARS-CoV-2 NSP15 Endoribonuclease, the compound that showed the best affinity parameter was gallagic acid with −9.3 kcal/mol, followed by amentoflavone (−9.1 kcal/mol), 7-O-galloylquercetin (−8.3 kcal/mol) and alpha-amyrin and rhamnosylisoorientin (both −8.1 kcal/mol) (Table 1). The plant metabolites also interacted (via hydrogen bonds, van der Walls interactions, and π-π stacking) with His250, an active site residue, and with neighboring residues (Figure 5). 

**Figure 6:**
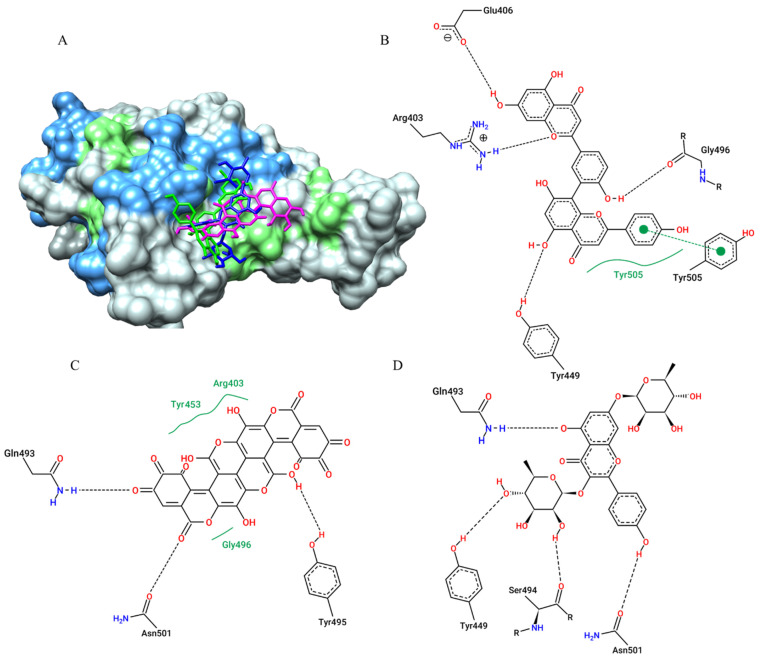
Surface representation of plant metabolites docking positions on the SARS-CoV-2 Spike protein (Spro) receptor-binding domain (RBD) active site, with amentoflavone (green), kaempferitrin (blue), and gallagic acid (magenta) (**A**). Contacts of SARS-CoV-2 Spike protein active site residues with amentoflavone (**B**), gallagic acid (**C**), and kaempferitrin (**D**) are depicted in a two-dimensional diagram. Dashed black lines represent hydrogen bonds, while full green lines represent van der Waals interactions, dashed green line represent π-π stacking.

For the SARS-CoV-2 Spike protein (Spro) receptor-binding domain (RBD), the compound that showed the best affinity parameter was amentoflavone (−8.7 kcal/mol), gallagic acid (−8.6 kcal/mol) and kaempferitrin (−8.0 kcal/mol) (Table 1). The plant metabolites also interacted (via hydrogen bonds, van der Walls interactions, and π-π stacking) with Tyr449 and Gln493 (active site residues), as well as with neighbouring residues involved in ACE-2 interaction (Figure 6). 

**Figure 7:**
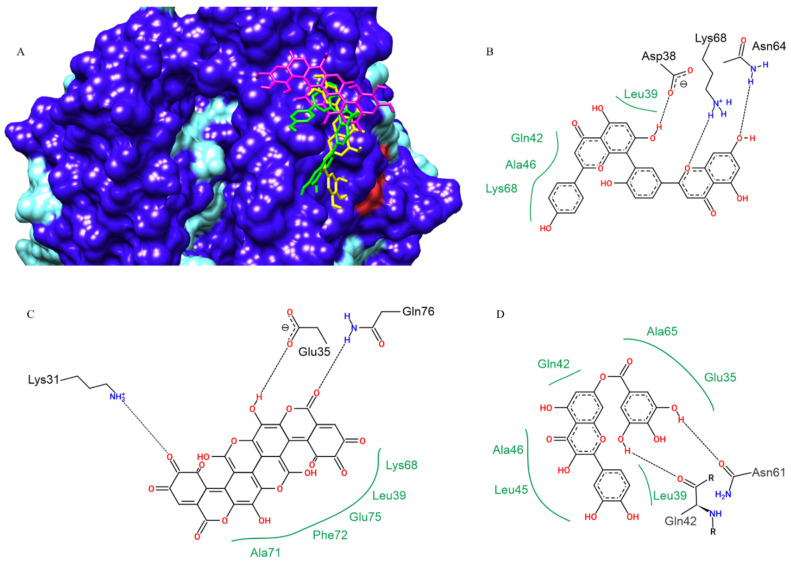
Amentoflavone (green), 7-O-galloylquercetin (yellow), and gallagic acid (magenta) docking positions on human ACE-2 (SARS-CoV-2 RBD spike protein binding site), in surface view (**A**). Contacts of SARS-CoV-2 Spike protein active site residues with amentoflavone (**B**), gallagic acid (**C**), and kaempferitrin (**D**) are depicted in a two-dimensional diagram. Dashed black lines indicate hydrogen bonds; full green lines indicate van der Waals interactions.

For human ACE-2, the compounds that showed the best affinity parameters were amentoflavone (−9.1 kcal/mol), gallagic acid (−8.9 kcal/mol) and 7-O-Galloylquercetin (−8.4 kcal/mol) in 5th (Table 1). The SARS-CoV-2 spike protein binds to human ACE-2 by a set of residues different from the functional active site of the enzyme. This is in the region encompassing residues Asp30, Asp32, Gln42, Tyr83, Lys353 and their respective neighbourhoods. The plant metabolites also performed their interactions (hydrogen bonds, van der Walls interactions) in this region, as well as with neighbouring residues used for interaction with ACE-2 (Figure 7).

### 2.2. In Vitro SARS-CoV-2 Spike-ACE2 Interaction Inhibitor Screening Assay

Due to the results obtained with molecular docking, amentoflavone and kaempferitrin were selected to be evaluated in vitro for their inhibitory potential on the interaction of SARS-CoV-2 Spro S1 receptor-binding domain (RBD) with ACE-2 through a specific kit. The probable activities of quercetin, luteolin, quercetin-7-O-glucoside (Quercimeritrin) and myricetin were also investigated; molecules that are already extensively studied in our research groups and that also showed considerable results in the molecular docking study. The 7-O-galloylquercetin and gallagic acid could not be experimentally evaluated because they were not found commercially, and we did not have conditions to proceed with the isolation and subsequent chemical analyses. Each of the tested molecules was evaluated at concentrations of 0.25; 0.5; 1; 2; 4; and 8 µg/mL. Under the test conditions, none of the selected molecules at any of the tested concentrations was able to inhibit the interaction of SARS-CoV-2 spike protein (Spro) receptor-binding domain (RBD) with ACE-2.

## 3. Discussion

Recently, after a period of relative epidemiological comfort due to the use of currently available vaccines, the number of new cases and deaths has again increased significantly in many countries, reactivating the concern about this virus and demonstrating that new types of vaccines and drugs are still urgently needed.

SARS-CoV-2 has a 29.9 kb RNA genome, still with 6 to 11 open reading frames (ORFs) [10]. The glycoprotein designated Spike (Spro) can bind to human angiotensin-converting enzyme 2 (ACE-2) and, from this interaction, viral adsorption is initiated. ACE-2 is present in the human upper respiratory tract and kidneys. Adsorption also depends on a serine protease, TMPRSS2 (transmembrane protease, serine 2) [35,36]. In addition to Spro, the virus has an envelope (E), nucleocapsid (N), and matrix (M) protein, as well as other accessory proteins that participate in modulating the innate host response [10,37].

In general, viral replication starts with the tropism and adsorption of the spike protein and human ACE-2 receptors. The intermembrane fusion proceeds, and the RNA genome is released into the cytoplasm of host cells. Initiated by two major non-structural polyproteins, pp1a and pp1ab, which are essential for viral replication and the formation of the replication complex (RTC), which in turn gives rise to virus structural proteins [38,39]. Virus assembly is completed with the formation of genomic RNA, nucleocapsid protein, and envelope protein. Disruption of the host cell membrane will lead to further infection [40,41]. The spike protein has two subunits, named S1 and S2, where S1 is responsible for tropism and range and S2 promotes intermembrane interaction [42].

The main studies are concentrated on the search for vaccines. However, these require time to be efficient and cannot be used in urgent and/or severe cases, which reinforces the urgent need for molecules that have the potential to be used as first choice drugs to combat the virus and the clinical symptomatology caused in the most urgent cases.

Computational predictions and the pharmacological properties of natural compounds have become promising against COVID-19. Using SARS-CoV-2 protein targets, many studies have focused on identifying possible phytochemical compounds with therapeutic potential against the virus through in silico assays [43,44,45]. Among the classes of natural compounds identified with antiviral action on SARS-CoV-2, we can highlight the flavonoids, alkaloids, and terpenes, including natural products from marine sources [46,47]. Sepay et al., [48] verified through DFT analysis, docking, and ADMET properties that benzylidenechromanones, a natural flavone-like compound, can inhibit proteins and important receptors for replication and transcription of SARS-CoV-2. Joshi et al., [45] found similar results after conducting an in silico screening to identify phytochemicals with antiviral properties. Ten compounds inhibited the Mpro, an essential enzyme from viral metabolism, and the ACE-2 enzyme, which the virus uses to enter the cell.

In our in silico study, amentoflavone, 7-O-galloylquercetin, kaempferitrin, and gallagic acid performed metabolically important chemical interactions such as hydrogen bonds, van der Walls interactions, and π-π stacking with active site amino acid residues and their neighbourhoods in the six targets evaluated, and thus were prioritized for being among the five best ranked compounds, demonstrating the best electronic affinity parameters in at least four of the six selected.

Amentoflavone, an apigenin-dimer, was evaluated in vitro against respiratory syncytial virus (RSV) showing great activity [49]. Significant activity has also been demonstrated against influenza A (subtypes H1N1 and H3N2) and B viruses, as well as moderate activity against herpes viruses (HSV-1 and HSV-2) [50], HSV-1 susceptible and acyclovir-resistant strains [51], and potential inhibition of replication of Coxsackie virus B3 (CVB3) [52], Hepatitis C virus (HCV) [53] and HIV-1 [54]. When evaluated in vitro against the SARS-CoV, amentoflavone was highly successful in inhibiting the SARS-CoV 3CLpro (syn. M^pro^) main protease, showing an IC_50_ value of 8.3 µM, being 35-fold more efficient than its monomer, apigenin. The docking result also showed the highest affinity parameters from amentoflavone with the SARS-CoV M^pro^ structure [55]. These results were according to our results (−8.7 kcal/mol) from docking between amentoflavone and SARS-CoV-2 (COVID-19) M^pro^, according to [56], the SARS-CoV-2 M^pro^ three-dimensional structure is highly similar to that of the SARS-CoV M^pro^ with 96% sequence identity.

Amentoflavone was also assessed in vitro at 40 µM against SARS-CoV with and without Triton X-100 to evaluate the influence of this non-ionic surfactant in their biological activity. When Triton X-100 was present, amentoflavone showed no antiviral activity, but it showed the highest activity without Triton use [44]. It was reported that dimethyl sulfoxide (DMSO) did not neutralize the activity of the molecule [57]. We believe that a possible explanation for this is that Triton X-100 can cleave amentoflavone, generating two apigenins, which, as observed by Ryu et al., [55], has no activity against SARS-CoV in low concentrations. Structural–activity relationships (SARs) also performed indicate that the absence of methoxy groups (that do not occur in amentoflavone) increases inhibitory activity against SARS-CoV 3CLpro, while the other tested molecules that present the referred groups in their structures were less potent, yielding inferior results to that of amentoflavone. This molecule also inhibits SARS-CoV-2 Mpro in a fluorescence-based Mpro substrate cleavage assay, with an IC_50_ of 8.6 µM and was also able to inhibit the activity of protein disulphide-isomerase (PDI), whose activity is essential for thrombus formation [58].

For the first time, amentoflavone was reported on *Arrabidaea chica* (Bignoniaceae) (syn. *Fridericia chica*) by [59]. According to the in silico results, inside *A. chica* metabolites, amentoflavone was the compound that showed the best affinity parameters between all metabolites, being suggested as the major molecule that efforts to *A. chica* antinociceptive and anti-inflammatory activity were found in this study. According to the authors, amentoflavone may inhibit the phospholipase A2 and cyclooxygenase pathways [60]. Concerning analgesic activity, amentoflavone acts as in vitro antagonists for kappa (*κ*), (mu) *μ*, and (delta) *δ* opioid receptors, being more than 10-fold selective for the *κ* over the *δ* opioid receptor [61]. When compared to other flavonoids, amentoflavone inhibited mast cell histamine secretion the most [62].

Amentoflavone also showed potential anti-diabetic activity by regulating glucose and lipid metabolism by decreasing levels of glucose, total cholesterol, triglyceride, low-density lipoprotein cholesterol (LDL-C) and glucagon, and increasing levels of high-density lipoprotein cholesterol (HDL-C) and insulin [63] in addition to promoting protective effects against cardiovascular dysfunction and liver damage in rats with induced metabolic syndrome. It was verified that these cardio and hepatoprotective effects are due to inhibition of the renin-angiotensin system and reduction of oxidative stress caused by amentoflavone [64]. Thus, we demonstrate that amentoflavone, besides the activity on SAR’s virus and activity against SARS-CoV-2, presents anti-nociceptive and anti-inflammatory activity at central and peripheral levels and antithrombotic activity, and may be considered a lead compound due to their potential therapeutically usefulness to eliminate SARS-CoV-2 from the organism of the patients as well as in the symptoms of acute disease.

We have previously reported through in silico studies the anti-inflammatory and analgesic activity of kaempferitrin, a kaempferol glycoside, which was identified in the hydroethanolic extract of the pollen collected by the stingless bee *Scaptogrinona aff postica* [19]. Kaempferitrin was experimentally evaluated about their potential analgesic and anti-inflammatory, with expressive results for both activities [65,66]. Furthermore, the inhibitory potential of kaempferitrin against lipopolysaccharide (LPS) and interferon (IFN)-gamma-induced nitric oxide (NO), and cytokines [tumour necrosis factor (TNF)-alpha and interleukin (IL)-12] in a dose-dependent manner was reported [67]. The potential activity of kaempferitrin on the central nervous system was assessed and evidenced that this molecule showed an antidepressant-like effect as a selective 5-HT1A agonist [68].

The high kaempferitrin activity against *Bacillus cereus* and *Enterococcus faecalis* was reported with minimum inhibitory concentration (MIC) activity values of 8.5 μg/mL and 3.9 μg/mL, respectively, besides presenting antimicrobial activity on *Shighella flexinerii*, *Salmonella typhimurium* and *Acinetobacter calcoaceticus* [69] and against *Artemisia salina* L. [70]. Regarding kaempferitrin’s anti-viral activity, this flavonoid showed potent activity against influenza A viruses H1N1, A/PR/8/34, and H3N2 [71]. Even with these activities against several microorganisms, kaempferitrin did not show mammalian toxicity, as it has a median lethal dose (LD_50_) > 2 g/kg in mice when administered intraperitoneal [72] and has not demonstrated acute toxicity or gastric damage to orally treated animals with 50 mg/kg of kaempferitrin [66].

Kaempferitrin also demonstrated a strong reducing effect on blood glucose levels in diabetic rats and stimulated glucose uptake as effectively as insulin in normal rat muscles [73]. Tzeng and co-authors [74] demonstrated a double effect of kaempferitrin, reporting that it improves insulin resistance by activating the classic insulin transduction pathway and increasing adiponectin secretion. The diuretic effect—and consequent antihypertensive effect of this molecule—is also reported, suggesting that it should be considered in the search for new treatments for renal and cardiovascular disorders [75]. These data reinforce that kaempferitrin is a lead compound for active drug research on COVID-19 because among the risk groups that can be more severely affected by COVID-19 are the diabetics who usually suffer severe consequences of this infection. Kaempferitrin presented data indicating that SARS-CoV-2 has a high potential to act on several targets of this organism, in addition to having anti-inflammatory activity, low toxicity to vertebrates, and anti-hyperglycemic activity, making it a promising alternative for research. 

Although it is commonly reported in the chemical composition of *Punica granatum* L. (pomegranate) and in *Terminalia* spp., studies on gallagic acid alone are rare. This molecule was reported to possess antioxidant activity (Dichloro-dihydro-fluorescein diacetate method), comparable with vitamin C, reducing the production of reactive oxygen species (ROS) and good antiplasmodium activity against *Plasmodium falciparum* and antimicrobial activity on *Pseudomonas aeruginosa*, *Cryptococcus neoformans* (also inhibiting growth), *Escherichia coli,* and methicillin-resistant *Staphilococcus aureus* [76].

As also gallagic acid, 7-O-galloylquercetin has few experimental studies regarding its bioactivity. However, it was investigated by Roubalová et al., [77] whose findings suggest that in vitro activation of Nrf2 in RAW264.7 cells is mediated by increased reactive oxygen species (ROS) production and activation of p38 mitogen-activated protein kinase (MAPKs) and increases the activity of antioxidant enzyme NAD(P)H: quinone oxidoreductase 1 (NQO1) and protein levels of heme oxygen 1 (HO-1) and Glutamate-cysteine ligase regulatory subunit (GCLM) in Hepa1c1c7 cells. We previously reported through an in silico screening study that both amentoflavone and 7-O-galloylquercetin (syn quercetin-o-gallate) showed large affinity parameters with the COX-2 structure, suggesting that the anti-inflammatory activity of *Arrabidaea chica* Verlot extract may be associated with the presence of these molecules in this plant [59].

The development of a vaccine is long, slow and requires very high financial costs, in addition to after administration of the vaccine, considerable time is needed until the complete establishment of the humoral response, which will provide immunity. The molecules presented in the present study are molecules found in many plant species worldwide, thus being sources of these compounds, but they can also be obtained synthetically without great difficulties, thus being able to be used in acute cases or in immediate need of an effective therapy against SARS-CoV-2.

More in-depth or robust assays involving SARS-CoV-2 are still not feasible in many laboratories due to structural and financial limitations, including the inability to guarantee the necessary biosafety conditions for researchers. 

The in vitro assay evaluates the potential of molecules to inhibit the interaction of SARS-CoV-2 Spro with human ACE-2. It is widely recognized that the principal route of cell invasion of SARS-CoV-2 into human cells occurs through the interaction of SARS-CoV-2 Spro with ACE-2, which is present in pneumocyte II-type and human kidney cells.

The assay performed allowed us to demonstrate that, although the in silico indication of interaction of plant metabolites with both Spro and ACE-2 is favourable and very likely, these compounds were unable to inhibit the interaction of Spro with ACE-2.

As can be seen from an analysis of the crystallographic structures of the Spro x ACE-2 complexes, the interaction between these structures is complex and involves dozens of amino acid residues in both. We hypothesize that the relatively small spatial volume of the compounds is not sufficient to prevent the electronic interaction between all residues of Spro with human ACE-2, thus maintaining the interaction even in the presence of the compounds. 

Despite the non-inhibition of the SARS-CoV-2 Spro interaction with ACE-2, these compounds should not be disregarded in studies for potential treatments for patients with COVID-19 as further more robust evaluations, such as: can these molecules interfere or prevent with the penetration of viral genetic material during Spro x ACE-2 interaction, thus preventing intracellular parasitism, or the activity on the other selected targets of SARS-CoV-2, such as main protease and RdRp that participate in the replication/transcription of SARS-CoV-2, are extremely necessary, since the indicative activity is relevant, the literature already reports activity of these molecules on other viruses, including the Coronaviridae family.

The use of natural products in the clinical routine of patients affected by COVID-19 is already a reality, where, in general, patients who received pharmacological treatments with natural products or those associated with synthetic drugs showed a much better clinical outcome than patients who received only treatment with traditional drugs, by delaying disease progression and reducing mortality rates [78,79,80,81,82].

Finding a bioactive chemical alone is a difficult task; developing novel therapeutic solutions is more difficult given the pressing need for new medications to be widely accessible worldwide. Even though there are various vaccines available to protect against SARS-CoV-2, additional therapies are still critically required to prevent the pandemic because many nations are unable to access any vaccine. Natural products are being researched as instruments to produce novel treatments since phytomedicine is more widely accepted by society than synthetic pharmaceuticals, mostly due to its excellent safety and the fact that its side effects tend to be less.

## 4. Materials and Methods

### 4.1. Choice and Preparation of the Structures of the Compounds

The 55 plant metabolites used in the present study were selected from our previous studies of anti-inflammatory activity [19,59,83,84,85] and were structurally schematized in three dimensions (3D) with the software GaussView 5.0.8 [86] and had their geometric and vibrational properties calculated (optimized) in vacuum at the density functional theory (DFT) level using the hybrid B3LYP functional combined with the 6–31 ++ G (d, p) basis with the Gaussian 09 software [87] to obtain the atomic and molecular electronic properties that correlate with possible biological activity.

### 4.2. Target Structures

The structures of macromolecule drug therapy targets of SARS-CoV-2 were used being: main protease (Mpro, 3CLpro) (PDB ID 6M03), RNA-dependent RNA polymerase (RDRP) (PDB ID 6M71), papain-like protease (PLpro) (PDB ID 6W9C), NSP15 Endoribonuclease (PDB ID 6VWW), SARS-CoV-2 RBD S1 Spike Protein (S Protein) and human angiotensin-converting enzyme 2 (ACE-2) (PDB ID 6M0J) available in the Protein Data Bank (PDB).

### 4.3. Molecular Docking

Docking was performed with the AutoDock Vina package [88]. The AutoDock Tools 1.5.7 module was used to prepare and analyse the computational calculations. After optimization, the structures of the plant metabolites were positioned in the central portion of the respective catalytic site of each selected target (Cys145 to Mpro, Asp618 to RdRp, Tyr264 to PLpro, His250 to NSP15, Gln493 to RDB Spro and Arg273 to ACE-2). Gasteiger charges and polar hydrogens, required for potential calculations, were added after removal of water molecules, drugs and/or artefacts from the target structures [89]. The targets macromolecules structures were kept rigid, while the ligands did not have their mobility restricted, remaining free. The size of the grid box was set to 22.5 Å for each axis. The number of modes was set to 50, and the exhaustiveness was set to 24. The conformations of the best interaction energy of the ‘ligand + receptor’ complexes identified in molecular docking were selected based on free energy of binding, by visual inspection and analysis of residues that best interacted with the ligand [19,20,90]. Molecular analyses and complex representations were obtained using the UCSF Chimera package [91] and PoseView [92].

### 4.4. In Vitro SARS-CoV-2 Spike-ACE2 Interaction Inhibitor Screening Assay

A specific SARS-CoV-2 Spike-ACE2 Interaction Inhibitor Screening Assay Kit (Item No. 502050; Cayman Chemical^®^, Ann Arbor, MI, USA) was used to assess the inhibitory potential of the recombinant SARS-CoV-2 Spro S1 RBD interaction with recombinant ACE-2. Amentoflavone, kaempferitrin, quercetin, luteolin, quercetin-7-O-glucoside (Quercimeritrin) and myricetin were obtained commercially (Sigma-Aldrich St. Louis, MO, USA; analytical grade 93–99%) and evaluated each at concentrations of 0.25; 0.5; 1; 2; 4, and 8 µg/mL. The test was prepared according to the manufacturer’s guidelines and read at 450 nm (Synergy™ H1, Biotek/AGILENT TECHNOLOGIES, Santa Clara, CA, USA).

## 5. Conclusions

The natural products are characterized as lead compounds in the anti-COVID19 drugs research since these substances have demonstrated the capacity to inhibit viral invasion and replication and modulate the immune-inflammatory response.

Computational protocols are important tools for the research of new therapeutic agents as they direct the search to more promising results. Our results show favourable interactions of amentoflavone, kaempferitrin, 7-O-galloylquercetin and gallagic acid with all the targets evaluated (Main Protease (Mpro or 3CL or MainPro), RNA-dependent RNA polymerase (RdRp), Papain-Like Protease (PLpro), NSP15 Endoribonuclease, Spike Protein (Protein S or Spro) and human Angiotensin-converting enzyme 2 (ACE-2).

Through the data obtained, we can demonstrate that although the indication of individual interaction of plant metabolites with both Spro and ACE-2 is very probable, the metabolites evaluated were unable to inhibit the interaction between these two structures. Despite this, these molecules still must be considered in the research of therapeutic agents for treatment of patients affected by COVID-19 since their activity on other targets and influence on the dynamics of viral infection during the interaction Spro x ACE-2 should be investigated.

In addition, there is robust literature indicating that these molecules have activity on several types of viruses and microorganisms, being mildly toxic to vertebrates and presenting activity on the complications of the disease, suggesting their potential that is worthy of further investigation.

## Figures and Tables

**Table 1:** Free binding energies values obtained by molecular docking of the 55 plant metabolites against SARS-CoV-2 main drug targets.

MainPro		RdRp		Papain-like Protease		NSP15 Endoribonuclease		Spike Protein		ACE-2	
Ligand	ΔGbind *	Ligand	ΔGbind *	Ligand	ΔGbind *	Ligand	ΔGbind *	Ligand	ΔGbind *	Ligand	ΔGbind *
Amentoflavone	−8.7	Amentoflavone	−9.4	Amentoflavone	−7.7	Gallagic acid	−9.3	Amentoflavone	−8.7	Amentoflavone	−9.1
7-O-Galloylquercetin	−8.6	Kaempferitrin	−9.3	Kaempferitrin	−7.5	Amentoflavone	−9.1	Gallagic acid	−8.6	Gallagic acid	−8.9
Kaempferitrin	−8.6	Gallagic acid	−9.0	7-O-Galloylquercetin	−7.4	7-O-Galloylquercetin	−8.3	Kaempferitrin	−8.0	Nicotinflorin	−8.5
Digalloylshikimic acid	−8.4	7-O-Galloylquercetin	−8.8	Myricitrin	−7.3	Alpha-amyrin	−8.1	Isoschaftoside	−7.7	Digalloylshikimic acid	−8.5
Gallagic acid	−8.2	Typhaneoside	−8.6	Suspensaside	−7.3	Rhamnosylisoorientin	−8.1	Vitexin	−7.6	7-O-Galloylquercetin	−8.4
Quercetin 7-O-glucoside	−8.1	Verbascoside/Acteoside	−8.5	Isoquercetrin	−7.1	Beta-amyrin	−8.0	Orientin	−7.5	Rutin	−8.2
Luteolin 7-galactoside	−8.0	Beta-amyrin	−8.3	Afzelin	−7.1	Ursolic acid	−8.0	Quercetrin	−7.4	Ursolic acid	−8.2
Quercetrin	−7.9	Rutin	−8.3	Beta-amyrin	−7.0	Isoorientin	−7.8	Myricetin	−7.4	Myricetin	−8.1
Rutin	−7.9	Myricetin	−8.3	Luteolin 7-galactoside	−7.0	Rutin	−7.8	7-O-Galloylquercetin	−7.3	Isoorientin	−8.1
Myricitrin	−7.8	Nicotinflorin	−8.2	Gallagic acid	−7.0	Digalloylshikimic acid	−7.7	Rutin	−7.3	Alpha-amyrin	−7.9
Nicotinflorin	−7.8	Alpha-amyrin	−8.1	Alpha-amyrin	−6.9	Ellagic acid	−7.7	Verbascoside/Acteoside	−7.3	Anthraquinone	−7.9
Rhamnosylisoorientin	−7.7	Rhamnosylisoorientin	−8.0	Digalloylshikimic acid	−6.9	Myricetin	−7.7	Isoorientin	−7.1	Myricitrin	−7.9
Luteolin	−7.7	Isoschaftoside	−8.0	Quercetin 7-O-glucoside	−6.8	Nicotinflorin	−7.6	Isovitexin	−7.1	Azadiradione	−7.8
Quercetin	−7.6	Digalloylshikimic acid	−7.9	Verbascoside/Acteoside	−6.8	Verbascoside/Acteoside	−7.6	Luteolin 7-galactoside	−7.1	Ellagic acid	−7.8
Isoquercetrin	−7.5	Isovitexin	−7.9	Orientin	−6.7	Anthraquinone	−7.5	Beta-amyrin	−6.9	Vismione D	−7.8
Myricetin	−7.5	Azadiradione	−7.8	Rutin	−6.7	Azadiradione	−7.5	Rhamnosylisoorientin	−6.9	Quercetrin	−7.7
Orientin	−7.4	Isoorientin	−7.8	Rhamnosylisoorientin	−6.6	Isovitexin	−7.5	Ononin	−6.9	Quercetin	−7.7
Ellagic acid	−7.3	Luteolin 7-galactoside	−7.8	Ononin	−6.6	Luteolin 7-galactoside	−7.5	Protocathecuic acid	−6.9	Luteolin 7-galactoside	−7.6
Anthraquinone	−7.2	Orientin	−7.8	Ursolic acid	−6.6	Quercetrin	−7.5	Typhaneoside	−6.9	Chrysoeriol	−7.6
Afzelin	−7.2	Isoquercetrin	−7.7	Vitexin	−6.6	Afzelin	−7.4	Ellagic acid	−6.8	Afzelin	−7.5
Vitexin	−7.2	Vitexin	−7.7	Isoorientin 7,3′-dimethyl ether	−6.5	Luteolin	−7.4	Quercetin 7-O-glucoside	−6.8	Kaempferitrin	−7.5
Diosmetin	−7.1	Quercetin 7-O-glucoside	−7.6	Isovitexin	−6.5	Orientin	−7.4	Alpha-amyrin	−6.7	Ononin	−7.5
Isoorientin 7,3′-dimethyl ether	−7.1	Quercetrin	−7.6	Nicotinflorin	−6.5	Diosmetin	−7.3	Afzelin	−6.7	Isovitexin	−7.5
Azadiradione	−7.0	Isoorientin 7,3′-dimethyl ether	−7.5	Quercetrin	−6.5	Ononin	−7.3	Vismione D	−6.7	Quercetin 7-O-glucoside	−7.4
Beta-amyrin	−7.0	Myricitrin	−7.5	Typhaneoside	−6.5	Vitexin	−7.3	Digalloylshikimic acid	−6.6	Rhamnosylisoorientin	−7.4
Isoorientin	−7.0	Ononin	−7.5	Quercetin	−6.4	Isoorientin 7,3′-dimethyl ether	−7.2	Isoorientin 7,3′-dimethyl ether	−6.5	Orientin	−7.4
Isovitexin	−7.0	Afzelin	−7.4	Azadiradione	−6.3	Carajurin	−7.1	Ursolic acid	−6.5	Kaempferol	−7.4
Ononin	−7.0	Ursolic acid	−7.3	Chrysoeriol	−6.2	Chrysoeriol	−7.1	Azadiradione	−6.4	Rhamnocitrin	−7.4
Ursolic acid	−7.0	β-sitosterol	−7.2	Ellagic acid	−6.2	Isoquercetrin	−7.1	Chrysoeriol	−6.4	Protocathecuic acid	−7.4
β-sitosterol	−6.9	Diosmetin	−7.0	Isoorientin	−6.2	Isoschaftoside	−7.1	Glucogallin	−6.4	Beta-amyrin	−7.3
Alpha-amyrin	−6.9	Ellagic acid	−7.0	Isoschaftoside	−6.2	Myricitrin	−7.1	Nicotinflorin	−6.4	Naringenin	−7.3
Kaempferol	−6.9	Glucogallin	−6.9	Luteolin	−6.2	Naringenin	−7.1	Myricitrin	−6.3	Luteolin	7.3
Rhamnocitrin	−6.9	Quercetin	−6.8	β-sitosterol	−6.1	Typhaneoside	−7.1	β-sitosterol	−6.2	Vitexin	−7.2
Chrysoeriol	−6.8	Chrysoeriol	−6.7	Anthraquinone	−6.1	Kaempferitrin	−7.0	Luteolin	−6.2	Diosmetin	−7.2
Glucogallin	−6.8	Kaempferol	−6.7	Diosmetin	−6.1	Quercetin	−7.0	Diosmetin	−6.1	Verbascoside/Acteoside	−7.1
Naringenin	−6.8	Protocathecuic acid	−6.7	Naringenin	−6.0	Kaempferol	−6.9	Isoquercetrin	−6.1	Isoorientin 7,3′-dimethyl ether	−7,0
5,7-Dimethoxyluteolin	−6.7	Anthraquinone	−6.6	Glucogallin	−5.9	Quercetin 7-O-glucoside	−6.9	Naringenin	−6.1	Isoquercetrin	−6.9
Verbascoside/Acteoside	−6.7	Luteolin	−6.6	Kaempferol	−5.9	Rhamnocitrin	−6.9	Quercetin	−6.1	β-sitosterol	−6.9
Vismione D	−6.6	Rhamnocitrin	−6.6	Protocathecuic acid	−5.9	Protocathecuic acid	−6.8	Rhamnocitrin	−6.1	Glucogallin	−6.9
Protocathecuic acid	−6.5	Naringenin	−6.5	Rhamnocitrin	−5.8	Vismione D	−6.7	Anthraquinone	−6.0	Isoschaftoside	−6.6
Isoschaftoside	−6.2	Carajurin	−6.3	5,7-Dimethoxyluteolin	−5.7	5,7-Dimethoxyluteolin	−6.6	Kaempferol	−6.0	5,7-Dimethoxyluteolin	−6.6
Carajurin	−6.0	5,7-Dimethoxyluteolin	−6.2	Carajurin	−5.6	Glucogallin	−6.5	5,7-Dimethoxyluteolin	−5.9	Carajurin	−6.5
Typhaneoside	−6.0	Vismione D	−6.0	Vismione D	−5.6	Caffeic acid	−6.2	Carajurin	−5.9	Beta-caryophyllene	−6.5
Caffeic acid	−5.5	Gallic acid	−5.9	Beta-caryophyllene	−5.5	β-sitosterol	−6.1	Gallic acid	−5.7	Elemol	−6.5
Gallic acid	−5.3	Caffeic acid	−5.4	Elemol	−5.3	Elemol	−5.8	Beta-caryophyllene	−5.5	Caffeic acid	−6.5
Beta-caryophyllene	−5.2	Elemol	−5.4	Thymol acetate	−5.3	Beta-caryophyllene	−5.7	Caffeic acid	−5.4	Cumaric acid	−6.2
Thymoquinone	−5.1	Beta-caryophyllene	−5.3	Beta-elemene	−5.2	Cumaric acid	−5.7	Elemol	−5.3	Typhaneoside	−6.1
Cumaric acid	−4.9	Thymol acetate	−5.2	Caffeic acid	−5.1	Carvacrol	−5.4	Beta-elemene	−5.1	Linoleic acid	−5.9
Elemol	−4.9	Thymoquinone	−5.2	Carvacrol	−4.9	Alpha terpineol	−5.3	Thymol acetate	−5.1	Carvacrol	−5.9
Beta-elemene	−4.8	Beta-elemene	−5.1	Cumaric acid	−4.9	Linolenic acid	−5.3	Thymoquinone	−5.1	Beta-elemene	−5.7
Carvacrol	−4.8	Alpha terpineol	−4.9	Gallic acid	−4.8	Thymol acetate	−5.3	Alpha terpineol	−5.0	Thymol acetate	−5.6
Linolenic acid	−4.8	Cumaric acid	−4.9	Alpha terpineol	−4.7	Beta-elemene	−5.2	Cumaric acid	−5.0	Linolenic acid	−5.6
Thymol acetate	−4.8	Carvacrol	−4.8	Thymoquinone	−4.6	Thymoquinone	−5.2	Carvacrol	−4.9	Alpha terpineol	−5.5
Linoleic acid	−4.7	Linoleic acid	−4.2	Linoleic acid	−4.4	Gallic acid	−5.1	Linoleic acid	−4.2	Gallic acid	−5.4
Alpha terpineol	−4.3	Linolenic acid	−4.2	Linolenic acid	−4.4	Linoleic acid	−4.7	Linolenic acid	−4.2	Thymoquinone	−5.4

## Data Availability

Data is contained within the article.
